# Parameter Determination and Ion Current Improvement of the Ion Current Sensor Used for Flame Monitoring

**DOI:** 10.3390/s21030697

**Published:** 2021-01-20

**Authors:** Hanqing Xu, Weijun Fan, Jianwei Feng, Peiliang Yan, Shuchan Qi, Rongchun Zhang

**Affiliations:** 1 National Key Laboratory of Science and Technology on Aero-Engines Aero-Thermodynamics, School of Energy and Power Engineering, Beihang University, Xueyuan Road, Beijing 100191, China; xhq6789@buaa.edu.cn (H.X.); fweij@buaa.edu.cn (W.F.); buaafjw@buaa.edu.cn (J.F.); yanpeiliang@buaa.edu.cn (P.Y.); 2 Research Institute of Aero-Engine, Beihang University, Xueyuan Road, Beijing 100191, China; shuchanq@buaa.edu.cn

**Keywords:** flame monitoring, ion current sensor, sensor parameter, Bunsen burner

## Abstract

Flame monitoring of industrial combustors with high-reliability sensors is essential to operation security and performance. An ion current flame sensor with a simple structure has great potential to be widely used, but a weak ion current is the critical defect to its reliability. In this study, parameters of the ion current sensor used for monitoring flames on a Bunsen burner are suggested, and a method of further improving the ion current is proposed. Effects of the parameters, including the excitation voltage, electrode area, and electrode radial and vertical positions on the ion current, were investigated. The ion current grew linearly with the excitation voltage. Given that the electrodes were in contact with the flame fronts, the ion current increased with the contact area of the cathode but independent of the contact area of the anode. The smaller electrode radial position resulted in a higher ion current. The ion current was insensitive to the anode vertical position but largely sensitive to the cathode vertical position. Based on the above ion current regularities, the sensor parameters were suggested as follows: The burner served as a cathode and the platinum wire acted as an anode. The excitation voltage, anode radial and vertical positions were 120 V, 0 mm, and 6 mm, respectively. The method of further improving the ion current by adding multiple sheet cathodes near the burner exit was proposed and verified. The results show that the ion current sensor with the suggested parameters could correctly identify the flame state, including the ignition, combustion, and extinction, and the proposed method could significantly improve the magnitude of the ion current.

## 1. Introduction

Monitoring of a flame’s state, including the ignition, combustion, and blowout, in combustors with simple, low-cost, and high-reliability flame sensors, is essential to the security and performance of thermal power systems such as industrial boilers, vehicle engines, and ground and aviation gas turbines [1,2]. The primary applications of flame sensors are ignition detection and blowout alert, which prevent the combustor from the explosion caused by fuel accumulation in the combustor [3,4,5,6]. Moreover, flame sensors are routinely used to monitor and avert abnormal combustions in order to achieve the operation requirement of stable, high-efficiency, and low-emission combustion [7,8,9]. 

Various attempts were performed to precisely and quickly detect the normal ignition, abnormal combustion, and accidental blowout in different combustors. Accompanying these attempts, flame sensing technologies based on flame characteristics, including heat release, pressure fluctuation, luminescence, composition variation, and production of ions and electrons, have been proposed [2]. Meanwhile, flame sensors based on thermoelectricity, piezo-electricity, photoelectricity, and electrochemistry were developed [1,10,11]. 

The most prevailing flame sensors are optical emission and absorption sensors. For example, ultraviolet flame sensors have been widely used in ground gas turbine combustors to monitor flame states. Docquier et al. adopted an intensified CCD camera to investigate the chemiluminescence of OH*, CH*, and C2* radicals in premixed methane/air flames and observed that the OH* and CH*/C2* radicals were suitable for monitoring lean and rich flames, respectively [12]. Ding et al. investigated the relationship between equivalence ratios and chemiluminescence intensities in CH_4_/air flames and verified that the CH*/OH* intensity ratio could be an equivalence indicator [9,13]. Hariharan and Mishra proposed a wavelet technique with the CH signature to sense dynamic flame stability, including a stable flame, flame liftoff, and main flame extinction, which could be applied in active control systems to avert lean flame blowout [14]. Tsai and Young used ultraviolet and multiband infrared technology to develop a multisensory-based fire alarm system [15]. Deguchi et al. used laser diagnostics, including laser-induced fluorescence (LIF), laser-induced breakdown spectroscopy (LIBS), and tunable diode laser absorption spectroscopy (TDLAS) to monitor the flame temperature and species concentration for controlling secondary air allocation and achieving a higher combustion efficiency [16]. Liu et al. developed a fan-beam tomographic sensor using TDLAS to monitor the temperature and gas concentration, and validation of the sensor exhibited good applicability for flame monitoring [17]. Later, Liu et al. developed an online and highly spatially resolved imaging system based on TDLAS tomography to monitor the dynamic behavior of swirling flames, which helped to better understand the lean blowout (LBO) mechanism [18,19].

Optical sensors are non-intrusive and have the merits of a fast response and high resolution. However, the complicated structure of the sensor composed of many elaborate optical components reduces the sensor’s flexibility, reliability, and maintainability, and the optical windows opened on the combustor reduce the reliability of the combustor structure. Thus, most of the optical flame sensors are usually applied in fundamental research in laboratory, rather than practical flame monitoring in industrial combustors, especially in aero-engines.

For the flame monitoring of industrial combustors working under high pressure, high temperature, and drastic vibration, intrusive flame sensors, such as thermocouples and pressure transducers, are widely used. K-type thermocouples, which are broadly used in ground and aviation gas turbines, are mounted at the turbine exit to monitor the exhaust gas temperature, from which the flame state could be inferred. High frequency dynamic pressure transducers are installed on the afterburners of some turbojet engines, for detecting flame ignition. Intrusive sensors are also often used in lab-scaled experiments. For instance, Gardiner examined the potential of using exhaust gas temperature thermocouples combined with an electronic signal processing method for alarming flame-out. Results from a GE J-85 combustor verified that the proposed thermocouple flame sensor could identify flame-outs and the response time was less than 100 ms [20]. Rolando et al. detected soot and nanoparticles in a diffusion ethylene flame by transient-thermocouple-based measurements. Their results demonstrated that the method was potent in detecting the particulate volume fraction [21]. Muruganandam et al. used a microphone to analyze the acoustic emission in a premixed swirl-stabilized combustor and observed the short duration, localized extinguishing, and re-ignition events as the flame approached blowout [22]. Nair and Lieuwen observed that low-frequency spectrum and intermittent fluctuation of the acoustic signal could be the precursor of a lean premixed flame in combustors with pilot, swirl, and bluff-body stabilizers, and proposed the acoustic method to monitor the precursor of LBO [23,24].

However, thermocouples are easily oxidized in high temperature flames and respond slowly to flames. Acoustic methods need the impulse line to transmit pressure, which complicates the sensor structure and reduces the pressure accuracy. 

An ion current sensor with a simple structure, low cost, fast response, and good maintainability provides an alternative possibility for practical flame monitoring. It has been commonly used for combustion diagnostics in lab-scaled combustors and active combustion control in industrial thermal systems. For example, Strandh et al. used the spark plug as ion current sensor to measure the ion current in homogeneous charge compression ignition (HCCI) combustion and proved that the ion current signal could be an excellent indicator of the combustion timing [25]. Yoshiyama and Tomita used the spark plug as an ion current probe to detect the combustion quality in a commercial spark ignition (SI) engine [26]. Chorpening et al. developed a combustion control and diagnostics sensor (CCADS) based on flame electrical properties for lean premixed gas turbine combustors. A CCADS has the capabilities of monitoring the flashback, equivalence ratio, and combustion instability [27]. Li et al. proposed the ion current sensing method to detect lean blowout in a pulse combustor [4]. They verified that the ion current sensing method could help to determine the LBO limit and avert an unexpected LBO, and observed that the ion current sensor was more sensitive to the LBO precursor than the pressure sensor. Chang et al. used the ion current signals to detect the LBO events for bluff-body-stabilized flames under a low Reynolds number [3]. 

Previous applications in different combustors during recent decades have verified that an ion current sensor with a central electrode can be conveniently and rigidly mounted in a combustor chamber without dramatically altering the chamber structure. The ion current signals can be a fast and reliable indicator for flame state and the relevant operation parameters, and the ion current sensor is a better choice for practical flame sensing provided that the central electrode is reasonably mounted. Nevertheless, ion current sensors have the intrinsic defect of producing weak ion current signals, which can be easily interfered with by other electronic devices and difficult to be collected by the acquisition system. In addition, the weak signals would seriously reduce the accuracy and reliability of the sensor for flame sensing. Therefore, for monitoring flame states, including the ignition, combustion, and extinction of practical flames, the parameters of an ion current sensor, especially the electrode installation positions, should be correctly determined to obtain an intense and stable signal during combustion, and in contrast, very weak signals during extinguishing. 

In this paper, we recommend the reasonable sensor parameters for an ion current sensor used in flame monitoring of a Bunsen burner, and a method for further improving the ion current is also proposed. Experiments were performed on a Bunsen burner since most of the practical flames consist of premixed and diffusion flames, and similar in combustion mechanism and flame structure to the flame on a Bunsen burner. Effects of the sensor parameters, including electrode polarity, excitation voltage, electrode area, and electrode radial and vertical positions, on the ion current were investigated first. Then, based on the regularities of the ion current, the reasonable sensor parameters for monitoring the flame’s state of ignition, combustion, and extinction were recommended. Meanwhile, the recommended parameters were testified in a wide operating range, from weak to strong combustion, by altering the propane volumetric flowrate. A method of adding multiple sheet cathodes near the burner exit to further improve the ion current during combustion was proposed, and the effectiveness of the method was validated in a wide operating range.

## 2. Experimental Investigation

### 2.1. Testing Apparatus

Experiments were performed with a partially premixed flame burning on a Bunsen burner and the flame ion current was detected by the ion current sensor. The raw data were collected by the data acquisition system.

#### 2.1.1. Bunsen Burner

**Figure 1:**
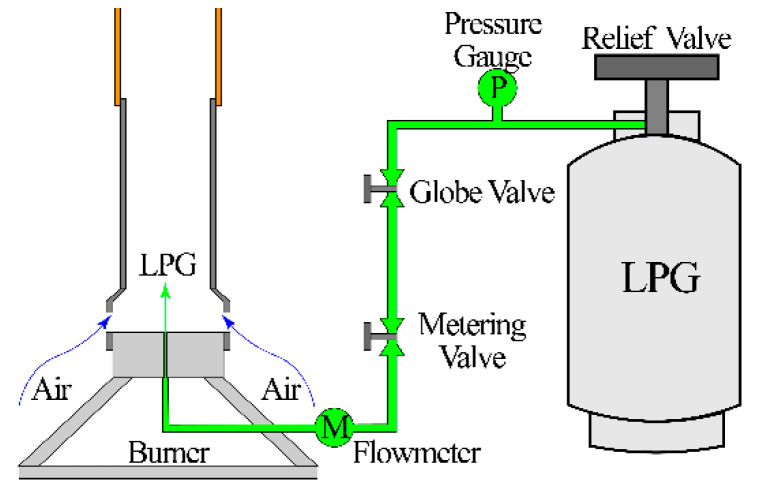
Schematic of the Bunsen burner.

The schematic of the Bunsen burner is sketched in Figure 1. The fuel, liquid propane gas (LPG), stored in a gas bomb, was injected into the burner through the 0.5 mm in diameter orifice drilled at the burner base. The gas bomb and the fuel injection orifice were connected by a rubber pipe, along which a relieve valve, pressure gauge, globe valve, metering valve, and volumetric flowmeter were mounted in sequence. The globe and metering valves were used to roughly and finely adjust the propane volumetric flowrate (${\dot{Q}}_{f}$), respectively. The ${\dot{Q}}_{f}$ was monitored by the flowmeter with an accuracy of 2.5%. The air entrained by the high-speed propane jet flowed into the tube through the air intake opened at the tube bottom and then premixed with propane in the tube. The air entrained by the high-temperature burnt gas diffused into the remaining propane along the flame front. Thus, a coupled flame, including the premixed and diffusion flames, could be constructed after successful ignition. The inner diameter of the exit was 10 mm.

#### 2.1.2. Ion Current Sensor

**Figure 2:**
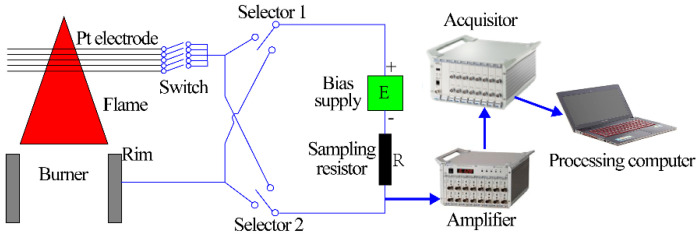
Schematic of the ion current acquisition system.

The schematic of the ion current sensor is depicted in the left part of Figure 2. The working circuit of the ion current sensor was a series circuit and mainly consisted of the burning flame, electrodes, and excitation power supply.

The core components of the sensor were two electrodes, the burner exit rim, by which the flame base was established and stabilized, and the platinum wire (ϕ = 0.5 mm), horizontally mounted above the burner exit and directly inserted into the flame. Obviously, the flame was in touch with the two electrodes in series. The DC excitation voltage ($U_{e}$) with a precision of 0.1% was outputted from the power supply and applied between the electrodes, propelling the charged particles in the flame to the electrodes. As a result, a current called ion current (I) was generated and it started to flow through the working circuit. A high-precision sampling resistor with a resistance (R) of 1 kΩ and precision of 0.01% was installed in series between the negative pole of the power supply and the electrode, for converting the ion current signal into a voltage signal that could be directly collected by the data acquisition system. The selectors placed between the electrodes and the power supply were used to switch the polarity of the voltage applied to the electrode, for studying the effect of voltage polarity on the ion current. The switch mounted between the platinum wires and the selectors was used to change the number ($n_{e}$) of the platinum wires jointed into the working circuit, for researching the effect of the electrode area ($S_{e}$) on the ion current.

#### 2.1.3. Data Acquisition System

The data acquisition system shown in the right part of Figure 2 was composed of the amplifier, acquisitor, and processing computer. The amplifier was used to amplify the voltage outputted from the sampling resistor. The acquisitor was used to collect the amplified voltage and transmit the voltage to the processing computer for analysis. The detailed performance parameters of the instruments are listed in Table 1.

### 2.2. Data Acquisition and Processing

The voltage signal from the ion current sensor was firstly amplified 100 times through the amplifier, then collected by the high-speed dynamic acquisitor, and finally recorded by the processing computer. Thus, the I could be inferred from the collected voltage ($U_{c}$) and R through the equation:

(1) $$I=\left(U_{c}\times 10^{-2}\right)/R$$

where the units of *I*, $U_{c}$, and *R* are A, V, and Ω, respectively.

For the average ion current, the sampling frequency (f) and sampling time (τ) at every test point were 1 kHz and 4.048 s, respectively. The measured value at the test point was specified by the arithmetic average of 4048 data points. For the dynamic ion current, f was 1 kHz.

## 3. Effects of the Sensor Parameters on the Flame Ion Current 

### 3.1. The Main Factors Affecting the Flame Ion Current

Factors having effects on the ion current are listed in Table 2. Note that these factors are collectively called sensor parameters since they represent the sensor structure and performance.

### 3.2. Effect of the Excitation Voltage on the Ion Current

**Figure 3:**
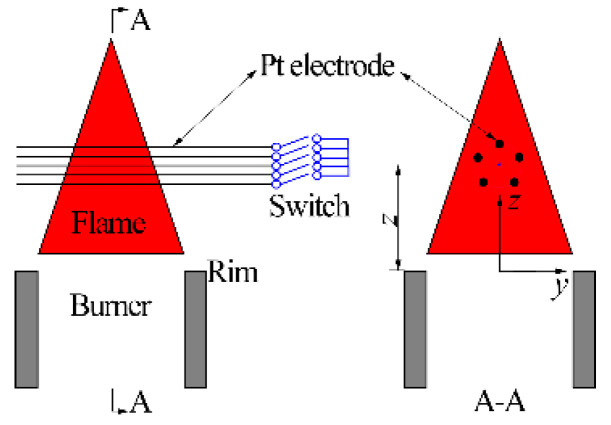
Schematic of the electrode structure for experiments of Factor 1.

Experiments for the effects of the excitation voltage on the ion current were conducted on the apparatus presented in Figure 3. Five parallel platinum wires covered by a porous corundum tube were horizontally mounted and placed through the entire flame along the transverse direction. They were uniformly arranged along the circumferential direction of a circle with a diameter of 5 mm. The *z* coordinate of the wire, representing the vertical distance between the wire and the burner exit, was specified as the electrode vertical position ($\Delta_{1}$). For experiments of Factor 1, $\Delta_{1}$ was the average vertical position of the 5 wires.

The experimental conditions for this trial are listed in Table 3. Since the polarity of the electrode may result in completely different regularities of the ion current, experiments for Factor 1 were divided into two groups: positive platinum wire/negative burner tube and negative platinum wire/positive burner tube.

**Figure 4:**
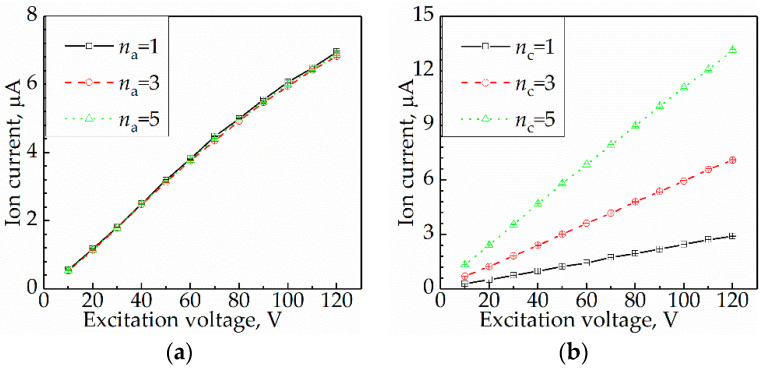
Effect of *U*_e_ on *I* at Δ_1_ = 5 mm: (**a**) platinum wire serving as an anode; (**b**) platinum wire serving as a cathode.

Results for the effect of $U_{e}$ on I are presented in Figure 4. Figure 4a displays the results when the platinum wire served as an anode, while Figure 4b shows the results when the platinum wire served as a cathode. The chain-dotted lines in each subgraph correspond to electrode number $n_{e}$ = 1, 3, 5.

#### 3.2.1. Platinum as an Anode, Burner as a Cathode

From Figure 4a, the three lines are nearly in mutual coincidence; it means that the regularities of I changing with $U_{e}$ were nearly identical under different anode areas ($S_{a}$). The straight lines with positive slopes (k) indicate that I almost grew linearly with *U*_e_. For instance, as *U*_e_ increased from 10 V to 120 V, *I* at *n*_a_ = 1 rose from 0.56 μA to 6.95 μA with *k* = 0.058 μA/V; I at $n_{a}$ = 3 grew from 0.53 μA to 6.82 μA with *k* = 0.057 μA/V; and *I* at *n*_a_ = 5 increased from 0.53 μA to 6.87 μA with k = 0.058 μA/V. It is clear to see that k is almost identical for all three $n_{a}$; this reveals that *S*_a_ has no effect on *I*.

#### 3.2.2. Platinum as a Cathode, Burner as an Anode

From Figure 4b, the three lines are similar to each other; it means the regularities of I changing with $U_{e}$ were similar under different cathode areas ($S_{c}$). The straight lines with positive k indicate that I went up linearly with $U_{e}$. For example, as $U_{e}$ increased from 10 V to 120 V, I at $n_{c}$ = 1 increased from 0.29 μA to 2.90 μA with k = 0.024 μA/V; I at $n_{c}$ = 3 grew from 0.69 μA to 7.08 μA with k = 0.058 μA/V; and *I* at *n*_c_ = 5 rose from 1.32 μA to 13.12 μA with k = 0.107 μA/V. As $n_{c}$ increased from 1 to 5, k rose from 0.024 μA/V to 0.107 μA/V. This reveals that $S_{c}$ has a great effect on I and I increased with $S_{c}$.

In summary, the flame ion current increased linearly with the excitation voltage.

#### 3.2.3. Analysis and Discussion

The working circuit of the ion current sensor can be simplified as a series circuit consisting of an excitation power supply ($U_{e}$), a flame resistor near the anode ($R_{a}$), and a flame resistor near the cathode ($R_{c}$). Thus, the ion current flowing through the circuit can be qualitatively deduced as follows.

(2) $$I=U_{e}{\left(R_{a}+R_{c}\right)}^{-1}$$

(3) $$R=\rho LS^{-1}$$

where R and ρ are the resistance and resistivity induced by the interaction between the flame and the electrode, respectively; L is the distance between the flame and the electrode; and *S* is the electrode area enclosed by the flame.

(4) $$\rho =\sigma^{-1},$$

(5) σ=μne,

where σ is the flame conductivity near the electrode; μ and n are the mobility and concentration of the charged particles in flame, respectively; and *e* is the unit charge [28].

For the electrodes inserted into the flame, the anode primarily attracts negative ions and electrons, whereas the cathode attracts mainly positive ions. Thus, the flame conductivity near the anode and cathode can be approximately expressed as

(6) $$\sigma_{a}\approx \mu_{e}n_{e}e,$$

(7) $$\sigma_{c}\approx \mu_{i}n_{i}e,$$

where subscript a and c denote the anode and the cathode, respectively; and subscript i and *e* denote the positive ions and the negative ions and electrons, respectively.

According to Equations (3)–(7), the $R_{a}$ and $R_{c}$ can be rewritten as

(8) $$R_{a}=L_{a}{\left(\mu_{e}n_{e}eS_{a}\right)}^{-1},$$

(9) $$R_{c}=L_{c}{\left(\mu_{i}n_{i}eS_{c}\right)}^{-1},$$

Thus, the ion current can be expressed as

(10) $$I=\frac{U_{e}}{L_{c}{\left(\mu_{i}n_{i}eS_{c}\right)}^{-1}+L_{a}{\left(\mu_{e}n_{e}eS_{a}\right)}^{-1}}.$$

From Equation (10), provided that the sensor parameters except for $U_{e}$ are determined, the I flowing through the working circuit of the ion current sensor is directly proportional to $U_{e}$. Consequently, the ion current increased linearly with the excitation voltage in the experiments, which justifies the fact that excitation voltage is an effective measure to improve the ion current.

### 3.3. Effect of Electrode Area on Ion Current

Experiments for the effect of the electrode area on the ion current were performed on the apparatus depicted in Figure 3. Experimental conditions for this trial are listed in Table 4.

**Figure 5:**
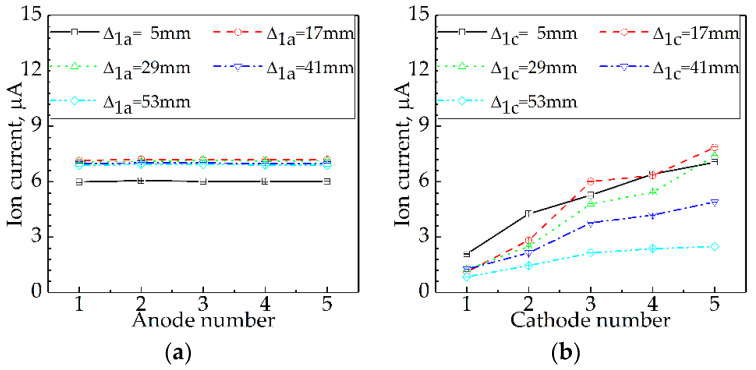
Effect of *S*_e_ on *I* at Δ_1_ = 5–65 mm: (**a**) effect of *S*_a_ on *I*; (**b**) effect of *S*_c_ on *I*.

Results for the effect of *S*_e_ on *I* are presented in Figure 5. Figure 5a displays the results for the effect of $S_{a}$ on I, while Figure 5b shows the results for the effect of $S_{c}$ on I. The chain-dotted lines in each subgraph correspond to $\Delta_{1}$= 5 mm, 17 mm, 29 mm, 41 mm, and 53 mm. $S_{e}$ was altered by changing $n_{e}$.

#### 3.3.1. Effect of Anode Area on Ion Current

From Figure 5a, the five curves are similar to each other; it means that the regularities of I changing with $S_{a}$ were similar under different $\Delta_{1a}$. The five horizontal lines reveal that as $n_{a}$ rose from 1 to 5, $S_{a}$ grew up, while I stayed constant. For instance, as $n_{a}$ increased from 1 to 5, I at $\Delta_{1a}$ = 5 mm, 17 mm, 29 mm, 41 mm, and 53 mm remained unchanged at about 6.03 μA, 7.20 μA, 7.03 μA, 7.00 μA, and 6.91 μA, respectively. This result is consistent with that in Section 3.2.1, which is that the anode area almost has no effect on the ion current.

#### 3.3.2. Effect of Cathode Area on Ion Current

From Figure 5b, the five curves are not exactly similar to each other, but with a similar tendency; it means that the relationships between *I* and *S*_c_ under different $\Delta_{1c}$ were qualitatively similar. The similar relationship is that I increased with *S*_c_ For example, as $n_{c}$ increased from 1 to 5, I at $\Delta_{1c}$ = 5 mm grew from 2.09 μA to 7.06 μA; I at $\Delta_{1c}$ = 29 mm rose from 1.26 μA to 7.40 μA; and I at $\Delta_{1c}$ = 53 mm increased from 0.85 μA to 2.49 μA. This result is also consistent with that in Section 3.2.2, which is that the ion current increased with the cathode area.

In summary, provided that the electrode was in contact with the flame fronts, the ion current remained constant with the anode area but increased with the cathode area.

#### 3.3.3. Analysis and Discussion

From Equation (10), I is directly proportional to $S_{a}$ and $S_{c}$, if the sensor parameters except for $S_{e}$ are determined. However, $n_{i}$ and $n_{e}$ in the flames can be considered at the same order of magnitude or nearly the same since the flame is electroneutral; that is,

(11) $$n_{e}\approx n_{i}$$

Moreover, $L_{a}S_{a}{}^{-1}$ and $L_{c}S_{c}{}^{-1}$ can be considered as the same or at the same magnitude order; that is,

(12) $$L_{a}S_{a}{}^{-1}\approx L_{c}S_{c}{}^{-1}.$$

In addition, $\mu_{i}$ and $\mu_{e}$ in the flames are greatly different from each other. $\mu_{i}$ is generally smaller than 1 ${\mathrm{cm}}^{2}V^{-1}s^{-1}$ since the positive ions are very heavy, while the $\mu_{e}$ is larger than 1000 ${\mathrm{cm}}^{2}V^{-1}s^{-1}$ since the negative ions and electrons are very light [28]; that is,

(13) $$\mu_{e}>>\mu_{i}.$$

Thus, according to Equations (8) and (9), $R_{a}<<R_{c}$, and $R_{a}$ can be neglected; it means that the electrical resistance in the flames is mainly concentrated in the vicinity of the cathode.

As a result, Equation (10) can be rewritten as

(14) $$I=U_{e}\mu_{i}n_{i}eS_{c}L_{c}{}^{-1}.$$

From Equation (14), provided that the electrodes are immersed into the flame, I is mainly subjected to $R_{c}$. The increasing of *S*_c_ can result in the reduction of $R_{c}$ and hence the rise of I. Consequently, the ion current remained constant with the anode area but increased with the cathode area in the experiments, and expanding the cathode area that is in contact with the flame can greatly amplify the flame ion current.

### 3.4. Effect of Electrode Radial Position on Ion Current

**Figure 6:**
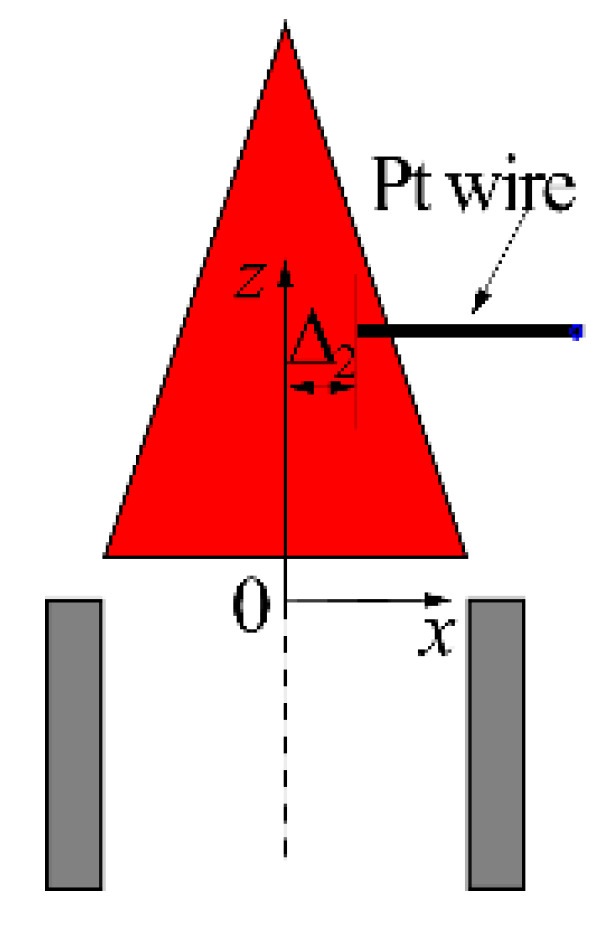
Schematic of the electrode structure for the experiments of Factor 3.

To understand the influence of the electrode radial position on the ion current, experiments were carried out on the apparatus displayed in Figure 6.

For minimizing the electrode interference with the flame, a single platinum wire was horizontally mounted, and the measurement area was limited in the right part of the flame. The electrode radial position was specified by $\Delta_{2}$, which is the electrode tip coordinate in the *x* direction. Experimental conditions for this trial are listed in Table 5.

**Figure 7:**
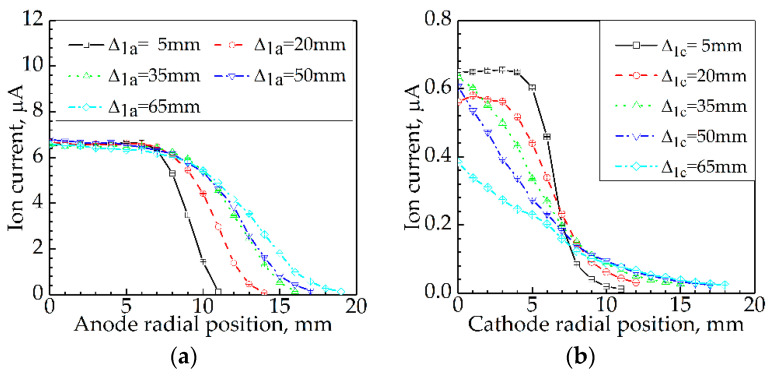
Effect of Δ_2_ on *I* at Δ_1_ = 5–65 mm: (**a**) effect of Δ_2a_ on *I*; (**b**) effect of Δ_2c_ on *I*.

Results of the effect of $\Delta_{2}$ on I are presented in Figure 7. Figure 7a displays the results for the effect of $\Delta_{2a}$ on I, while Figure 7b shows the results for the effect of $\Delta_{2c}$ on I. The chain-dotted lines in each subgraph correspond to $\Delta_{1}$ = 5 mm, 20 mm, 35 mm, 50 mm, and 65 mm.

#### 3.4.1. Effect of Anode Radial Position on Ion Current

According to the curve shapes, the five curves in Figure 7a can be divided into two groups. Group 1 includes the curves for $\Delta_{1a}$ = 5 mm, 20 mm, and 35 mm, and Group 2 contains the curves for $\Delta_{1a}$ = 50 mm and 65 mm. In each group, the regularities of the ion current changing with the anode radial position are similar to each other.

For Group 1, the curve shape, a nearly horizontal line followed by a monotonous declining curve, informs that as $\Delta_{2a}$ increased, I kept constant first, then gradually decreased to 0. For instance, at Δ_1a_ = 5 mm, with $\Delta_{2a}$ increasing from 0 mm to 6 mm, I remained constant at about 6.59 μA; when $\Delta_{2a}$ > 6 mm, I decreased with $\Delta_{2a}$. At $\Delta_{1a}$ = 20 mm, with $\Delta_{2a}$ growing from 0 mm to 6 mm, I stayed unchanged at about 6.59 μA; when $\Delta_{2a}$ > 6 mm, I reduced with $\Delta_{2a}$. At $\Delta_{1a}$ = 35 mm, with $\Delta_{2a}$ rising from 0 mm to 7 mm, I kept stable at about 6.49 μA; when $\Delta_{2a}$ > 7 mm, I declined with $\Delta_{2a}$.

For Group 2, the curve shape is an oblique line with a very small gradient followed by a monotonous declining curve with a larger gradient; this informs that as $\Delta_{2a}$ increased, I reduced slowly first, and then decreased to 0 more quickly. For example, at $\Delta_{1a}$ = 50 mm, with $\Delta_{2a}$ increasing from 0 mm to 8 mm, I dropped slowly from 6.79 μA to 6.16 μA with a smaller average gradient of 0.08 μA/mm; when $\Delta_{2a}$ > 8 mm, I decreased quickly with $\Delta_{2a}$ and the larger average gradient was 0.66 μA/mm. At $\Delta_{1a}$ = 65 mm, with Δ_2a_ rising from 0 mm to 8 mm, *I* decreased slowly from 6.58 μA to 6.04 μA with the lower average gradient of 0.07 μA/mm; when $\Delta_{2a}$ > 8 mm, I reduced rapidly with $\Delta_{2a}$ and the higher average gradient was 0.54 μA/mm.

Obviously, as the anode radial position increased, there was a critical position ($\Delta_{2\mathrm{at}}$) from which the ion current transformed from being constant to declining for the curves in Group 1, and transformed from a slow to rapid descent for the curves in Group 2. The $\Delta_{2\mathrm{at}}$ at $\Delta_{1a}$ = 5 mm, 20 mm, 35 mm, 50 mm, and 65 mm were 6 mm, 6 mm, 7 mm, 8 mm, and 8 mm, respectively.

In summary, if $\Delta_{2a}$ < $\Delta_{2\mathrm{at}}$, the ion current would stay constant or decrease slowly with $\Delta_{2a}$, and decrease quickly otherwise.

#### 3.4.2. Effect of Cathode Radial Position on Ion Current

According to the curve shapes, the curves in Figure 7b also can be sorted into two groups as in Figure 7a. Group 1 includes the curve for $\Delta_{1c}$ = 5 mm and Group 2 contains the other 4 curves for $\Delta_{1c}$ = 20 mm, 35 mm, 50 mm, and 65 mm. In each group, the regularities of the ion current changing with the cathode radial position are similar to each other.

For Group 1, the curve consists of a nearly horizontal line and a monotonous declining curve; this qualitatively informs that as $\Delta_{2c}$ increased, I was constant first and then decreased subsequently. In quantitative terms, at $\Delta_{1c}$ = 5 mm, with $\Delta_{2c}$ increasing from 0 mm to 4 mm, I remained constant at about 0.65 μA; when $\Delta_{2c}$ > 4 mm, *I* decreased with Δ_2c_.

For Group 2, the curve shape shows a monotonous declining curve, which informs that as $\Delta_{2c}$ increased, I reduced markedly. For example, at $\Delta_{1c}$ = 20 mm, as $\Delta_{2c}$ grew from 0 mm to 12 mm, *I* reduced from 0.57 μA to 0.03 μA; at $\Delta_{1c}$ = 50 mm, as $\Delta_{2c}$ increased from 0 mm to 17 mm, *I* dropped from 0.61 μA to 0.03 μA.

In summary, a smaller $\Delta_{2c}$ led to a larger I, and the deeper the cathode was inserted into the flame, the greater *I* would be captured.

#### 3.4.3. Analysis and Discussion

**Figure 8:**
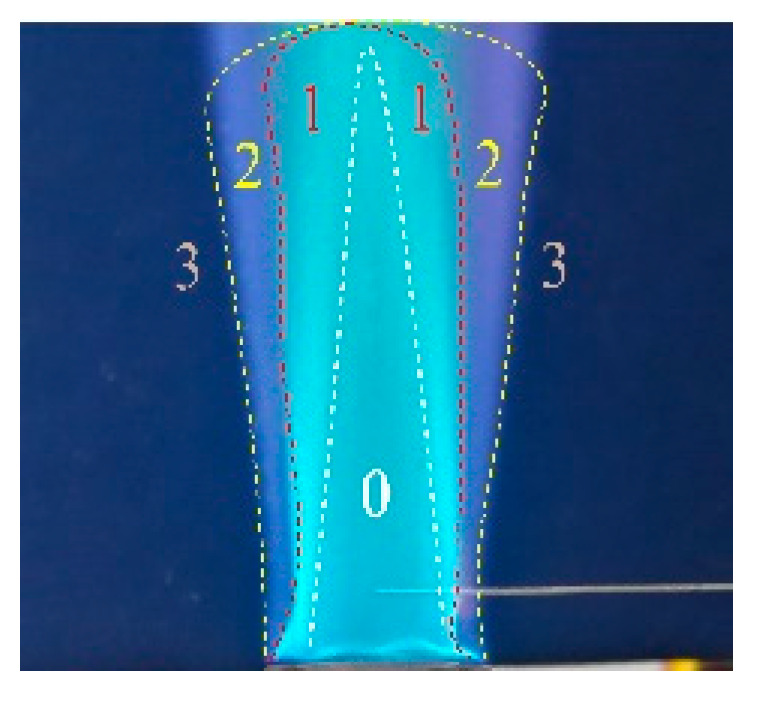
Zonal division of the Bunsen flame.

In order to further understand the regularities of *I* changing with $\Delta_{2}$, photos of the flame and platinum wire were taken. Figure 8 presents the photo of the Bunsen flame, in which a platinum anode was inserted under ${\dot{Q}}_{p}$ = 100 L/h. In this photo, the flame was divided into 4 zones numbered 0 to 3, respectively. Zone 0 is the premixing zone without combustion. Zone 1, where the premixed flame front with intense chemical reactions of combustion, contains a large amount of charged particles. Zone 2 is the diffusion combustion area, and contains a number of charged particles. Zone 3 is the burnt gas region.

The effects of $\Delta_{2}$ on I can be explained by the equations in Section 3.2.3. Since ${\dot{Q}}_{p}$ was constant in all experiments, the flame was steadily anchored at the burner exit and the electrical resistance of the flame near the burner exit was almost constant. Thus, according to Equation (2), the I variation was mainly subjected to the electrical resistance of the flame near the platinum wire.

**Figure 9:**
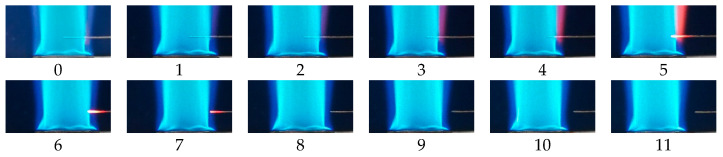
Flame photos at Δ_2a_ = 0–11 mm under Δ_1a_ = 5 mm. The numbers below each image represent the values of Δ_2a_.

For the effect of $\Delta_{2a}$ on I, the platinum wire served as the anode and the variation of I was subjected to $R_{a}$. Figure 9 shows the flame photos of $\Delta_{2a}$ = 0–11 mm at $\Delta_{1a}$ = 5 mm. From the figure, when $\Delta_{2a}$ = 0–6 mm, the anode was immersed in the flame; in comparison with $R_{c}$, $R_{a}$ was much smaller and can be neglected; thus, I was nearly constant with $\Delta_{2a}$. For $\Delta_{2a}$ = 7–11 mm, as the anode was gradually moved out of the flame, $S_{a}$ decreased and the gap length ($D_{\mathrm{af}}$) between the anode tip and flame fronts increased; this $D_{\mathrm{af}}$ would dramatically lower $\mu_{e}$. Thus, as $\Delta_{2a}$ increased, $S_{a}$ and $\mu_{e}$ both declined, which resulted in the rise of $R_{a}$ and the drop of I. In addition, it can be seen that $\Delta_{2\mathrm{at}}$ at $\Delta_{1a}$ = 5 mm was equal to $\Delta_{2a}$ = 6 mm, where the anode tip was critically in touch with the premixed flame front.

**Figure 10:**
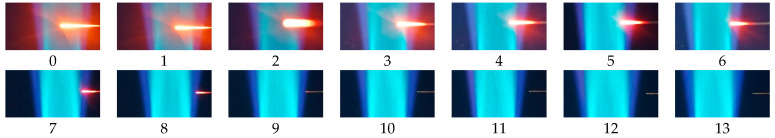
Flame photos at Δ_2a_ = 0–13 mm under Δ_1a_ = 20 mm. The numbers below each image represent the values of Δ_2a_.

**Figure 11:**
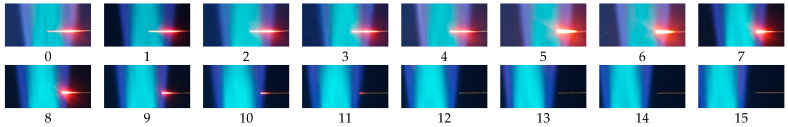
Flame photos at Δ_2a_ = 0–15 mm under Δ_1a_ = 35 mm. The numbers below each image represent the values of Δ_2a_.

The flame photos for $\Delta_{1a}$ = 20 mm and 35 mm are displayed in Figure 10 and Figure 11, respectively. It can be seen that, at such positions, $\Delta_{2\mathrm{at}}$ was located at $\Delta_{2a}$ = 6 mm and 7 mm, respectively. As $\Delta_{2a}$ < $\Delta_{2\mathrm{at}}$, the anode was immersed into the premixed flame front and I was constant. As $\Delta_{2a}$ > $\Delta_{2\mathrm{at}}$, the anode tip was moved out of the premixed flame front and *I* decreased with Δ_2a_.

**Figure 12:**
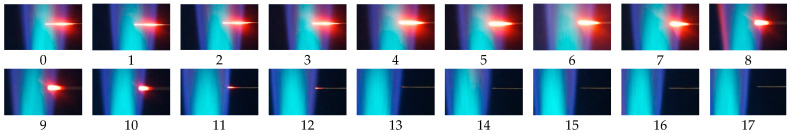
Flame photos at Δ_2a_ = 0–17 mm under Δ_1a_ = 50 mm. The numbers below each image represent the values of Δ_2a_.

**Figure 13:**
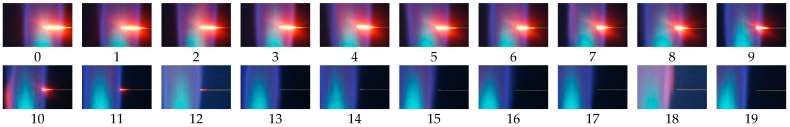
Flame photos at Δ_2a_ = 0–19 mm under Δ_1a_ = 65 mm. The numbers below each image represent the values of Δ_2a_.

Figure 12 and Figure 13 show the flame photos for $\Delta_{1a}$ = 50 mm and 65 mm. From these photos, the turbulent premixed flame front was unstable. In such case, the deeper the anode was immersed into the flame, the greater the probability that the anode would be in contact with the flame. When $\Delta_{2a}$ ≤ 8 mm, the anode was immersed inside the flame front, as a result, within the same duration of time, both the average $S_{a}$ and $\mu_{e}$ decreased with $\Delta_{2a}$. This resulted in the increase of $R_{a}$ and the gentle decrease of I with $\Delta_{2a}$. When $\Delta_{2a}$ > 8 mm, as the anode was gradually moved away from the flame front, $S_{a}$ decreased but $D_{\mathrm{af}}$ grew, which resulted in the rise of $R_{a}$ and the drop of I.

For the effect of $\Delta_{2c}$ on I, the platinum wire served as a cathode. Since $R_{a}$ was much smaller than $R_{c}$, the $R_{a}$ in this trial can be neglected. Therefore, I was only subjected to $R_{c}$. Flame photos for this experiment are the same as Figure 9, Figure 10, Figure 11, Figure 12 and Figure 13.

For $\Delta_{1c}$ = 5 mm, when $\Delta_{2c}$ < 4 mm, the cathode was inserted into the flame and the front part of the cathode was surrounded by the cold gas exhausted from the burner tube. Since the cold gas impeded the collection of positive ions by the cathode, the variation in the cooled part of the cathode had no effect on $R_{c}$. Therefore, I remained nearly constant with $\Delta_{2c}$. When $\Delta_{2c}$ > 4 mm, the cathode was removed away from the cold gas. As $\Delta_{2c}$ increased, $S_{c}$ decreased and the distance ($D_{\mathrm{cf}}$) between the cathode tip and the flame fronts increased, which led to the increase in $R_{c}$. As a result, I reduced with $\Delta_{2c}$.

For $\Delta_{1c}$ = 20–65 mm, since the flame fronts expanded with $\Delta_{1c}$, the effect of cold gas on the collection of positive ions by the cathode was very slight; hence, as $\Delta_{2c}$ increased, both $S_{c}$ and $\mu_{a}$ decreased and $R_{c}$ rose. As a result, I declined with $\Delta_{2c}$.

It can be concluded that putting the electrodes adequately in contact with the flame, particularly with the premixed flame front, is an effective measure to enhance the flame ion current.

### 3.5. Effect of Electrode Vertical Position on the Ion Current

Experiments for the effect of the electrode vertical position on the ion current were carried out on the apparatus displayed in Figure 6. The experimental conditions for this trial are listed in Table 6.

**Figure 14:**
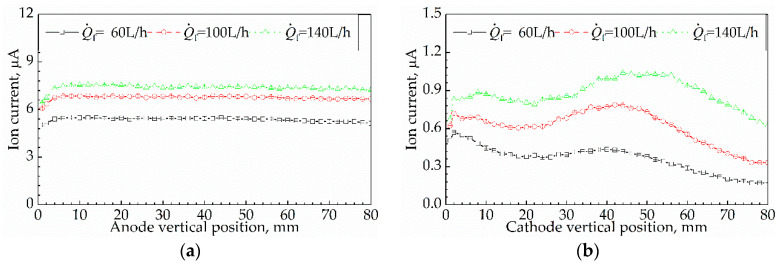
Effect of Δ_1_ on *I* at ${\dot{Q}}_{f}$ = 60–140 L/h: (**a**) effect of Δ_1a_ on *I*; (**b**) effect of Δ_1c_ on *I*.

Results for the effect of $\Delta_{1}$ on I are presented in Figure 14. Figure 14a displays the results for the effect of $\Delta_{1a}$ on I, while Figure 14b shows the results for the effect of $\Delta_{1c}$ on I. The chain-dotted lines in each subgraph correspond to ${\dot{Q}}_{f}$ = 60 L/h, 100 L/h, and 140 L/h.

#### 3.5.1. Effect of Anode Vertical Position on Ion Current

From Figure 14a, the shapes of the three curves are similar to each other; it means that the regularities of *I* changing with $\Delta_{1a}$ at ${\dot{Q}}_{f}$ = 60 L/h, 100 L/h, and 140 L/h were similar. The shape of the curves reveals that I increased with Δ_1a_, ranging from 1 mm to 6 mm, while remaining nearly constant with Δ_1a_, ranging from 6 mm to 80 mm. For instance, at ${\dot{Q}}_{f}$ = 60 L/h, as $\Delta_{1a}$ rose from 1 mm to 6 mm, I grew from 5.04 μA to 5.45 μA; when $\Delta_{1a}$ > 6 mm, I kept constant at 5.38 μA. At ${\dot{Q}}_{f}$ = 100 L/h, as $\Delta_{1a}$ rose from 1 mm to 6 mm, I increased from 6.07 μA to 6.84 μA; when $\Delta_{1a}$ > 6 mm, I was constant at 6.78 μA. At ${\dot{Q}}_{f}$ = 140 L/h, as $\Delta_{1a}$ rose from 1 mm to 6 mm, I increased from 6.42 μA to 7.48 μA; when $\Delta_{1a}$ > 6 mm, I kept constant at 7.41 μA. In conclusion, if $\Delta_{1a}$ > 6 mm, I remained constant and was insensitive to $\Delta_{1a}$.

#### 3.5.2. Effect of Cathode Vertical Position on Ion Current

From Figure 14b, the shapes of the three curves are also similar to each other, which indicates that the regularities of *I* changing with $\Delta_{1c}$ at ${\dot{Q}}_{f}$ = 60 L/h, 100 L/h, and 140 L/h were similar. The erratic wavy curve reveals that, as $\Delta_{1c}$ rose from 1 mm to 80 mm, I fluctuated in a wavy manner. For example, at ${\dot{Q}}_{f}$ = 100 L/h, as $\Delta_{1c}$ rose from 1 mm to 2 mm, I grew from 0.62 μA to 0.72 μA; when 2 mm < $\Delta_{1c}$ < 18 mm, I reduced from 0.72 μA to 0.61 μA with $\Delta_{1c}$; when 18 mm < $\Delta_{1c}$ < 42 mm, I increased from 0.61 μA to 0.79 μA with $\Delta_{1c}$; when 42 mm < $\Delta_{1c}$ < 80 mm, I dropped from 0.79 μA to 0.33 μA with $\Delta_{1c}$. In conclusion, I fluctuated with $\Delta_{1c}$ and was very sensitive to $\Delta_{1c}$.

#### 3.5.3. Analysis and Discussion

For the effect of $\Delta_{1a}$ on I, the platinum wire served as an anode and the variation in I was subjected to $R_{a}$. When $\Delta_{1a}$ = 1 mm, part of the anode was positioned in the dead space between the flame base and the burner exit, and disconnected with the flame base. As a result, $S_{a}$ was very small. Moreover, the cooling effects from the burner rim and cold gas on the flame and the anode led to the lower flame temperature and larger distance between the flame and the anode. It resulted in both $n_{e}$ and $\mu_{e}$ being much smaller than that in flames at another $\Delta_{1a}$. Consequently, when $\Delta_{1a}$ = 1 mm, $R_{a}$ increased to the maximum for $\Delta_{1a}$ = 1–80 mm and correspondingly I reached the minimum.

As for $\Delta_{1a}$ = 2–6 mm, the anode was out of the dead space and adequately in contact with the flame, and the cooling effects of cold gas and burner rim on the flame and anode were both weakened compared to $\Delta_{1a}$ = 1 mm. Therefore, $n_{e}$ and $\mu_{e}$ at $\Delta_{1a}$ = 2–6 mm were both higher, which resulted a smaller $R_{a}$; hence, a larger *I*. In addition, since the cooling effects were gradually weakened with $\Delta_{1a}$, I increased with $\Delta_{1a}$ ranging from 2 mm to 6 mm.

As for $\Delta_{1a}$ = 7–80 mm, the cooling effects nearly vanished, which led to the larger $n_{e}$ and $\mu_{e}$. Therefore, $R_{a}$ was smaller and I was higher. Since $R_{a}$ was so much smaller than $R_{c}$ that it could be negligible, I remained nearly constant at $\Delta_{1a}$ = 7–80 mm.

For the effect of $\Delta_{1c}$ on I, the platinum wire served as a cathode. Since $R_{a}$ was much smaller than $R_{c}$ and could be neglected, I was subjected only to $R_{c}$. Therefore, according to Equations (9) and (14), I was directly proportional to $n_{i}$.

**Figure 15:**
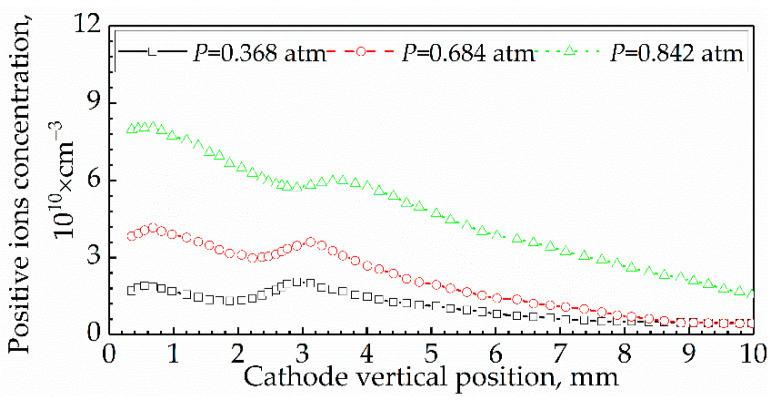
Profiles of the total concentration of positive ions in propane/air flames.

Figure 15 shows the previous results of the total concentration profiles of positive ions in propane/air flames along the height above a Bunsen burner [28]. The fuel equivalence ratio was 1.43; the burner exit diameter was 80 mm; and the volumetric gas flowrate was 450 L/h. In our experiment, the I profiles along $\Delta_{1c}$ represents the $n_{i}$ profile along the $\Delta_{1c}$ and is similar to the results in Figure 15. It can be inferred that the $n_{i}$ profiles along $\Delta_{1c}$ in our experiment are reasonable and conform to reality since the operation conditions in our experiment were similar to that in the experiment corresponding to Figure 15.

## 4. Determination of the Sensor Parameters for Flame Monitoring

### 4.1. Determination of the Sensor Parameters

The parameters of the ion current sensor must be determined before the sensor is applied to practical engineering. As for the sensor used for flame monitoring, the sensor parameters that are corresponding to the maximum ion current are recommended to be selected. Based on the regularities of the ion current changing with each sensor parameter described in the previous context, the ideal sensor parameters are suggested as follows.

- *U*_e_ = 120 V

Since *I* increased with *U*_e_, *U*_e_ for the ion current sensor should be set as 120 V, which was the maximum voltage of the DC power supply in the experiment.

- The burner rim served as a cathode, while the platinum wire acted as an anode.

In terms of $S_{e}$, I increased with $S_{c}$ and independent of $S_{a}$. Since the area of the burner rim was larger than that of the single platinum wire, the burner rim should be equivalent to a cathode, whereas the platinum wire should serve as an anode. 

In terms of $\Delta_{2}$, if the electrode was immersed into the flame, I was nearly constant for a different $\Delta_{2a}$, but decreased with $\Delta_{2c}$. Thus, making the cathode steadily in contact with the flame with a larger area could improve the magnitude of the ion current. Since the burner rim was very close to the flame base under various ${\dot{Q}}_{f}$ throughout, and the area of the burner rim was larger than that of the platinum wire, the burner rim should serve as the cathode, while the platinum wire should act as the anode.

In terms of $\Delta_{1}$, if $\Delta_{1}$ was more than 6 mm, *I* fluctuated with $\Delta_{1c}$ but was independent with $\Delta_{1a}$. Thus, *I* was sensitive to $\Delta_{1c}$ but insensitive to $\Delta_{1a}$. Since the distance between the burner rim and the flame base was nearly stable under various ${\dot{Q}}_{f}$, the burner rim should be a cathode, while the single platinum should serve as an anode.

In summary, the burner rim should be selected as the cathode, while the single platinum should be selected as the anode.

- Δ_2a_ = 0 mm.

When the anode radial position was 0 mm, the anode was adequately in contact with the flame fronts and I was stronger and more stable. Therefore, $\Delta_{2a}$ is recommended to be set as 0 mm.

- Δ_1a_ = 6 mm.

*I* increased with $\Delta_{1a}$ = 1–6 mm, and kept constant at $\Delta_{1a}$ = 6–80 mm. Consequently, to ensure that *I* is high and stable, $\Delta_{1a}$ should be selected as 6–80 mm. In this paper, $\Delta_{1a}$ was set as 6 mm.

### 4.2. Sensor Verification for Flame Monitoring

**Figure 16:**
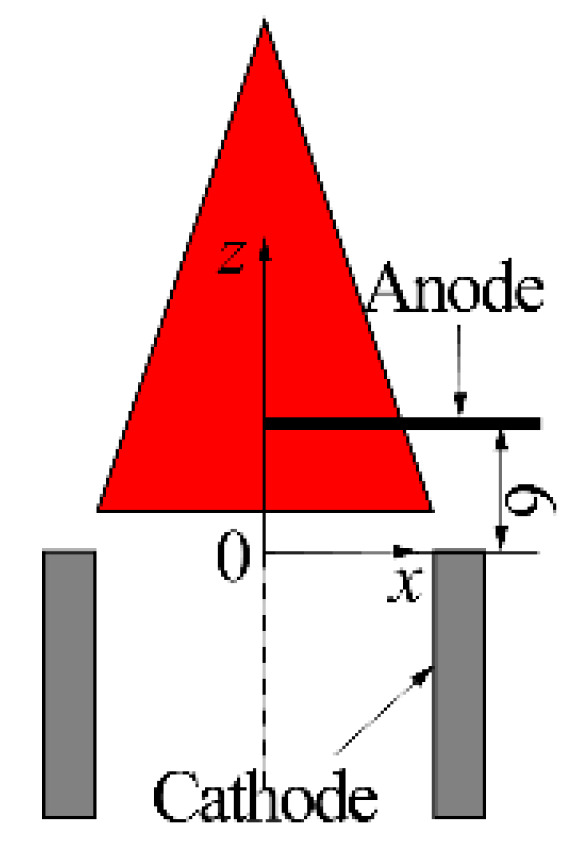
Schematic of the electrode structure with the recommended parameters.

**Figure 17:**
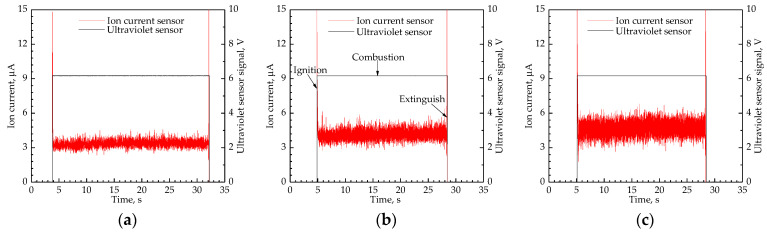
Ion current signals during flame monitoring: (**a**) ${\dot{Q}}_{f}$ = 50 L/h; (**b**) ${\dot{Q}}_{f}$ = 100 L/h; (**c**) ${\dot{Q}}_{f}$ = 150 L/h.

To verify the legitimacy of the sensor parameters determined in Section 4.1, the ion current sensor with the determined parameters was used for flame monitoring (ignition, combustion, and extinction) on a Bunsen burner. The determined sensor structure is depicted in Figure 16 and the I signals collected under ${\dot{Q}}_{f}$ = 50–150 L/h are displayed in Figure 17. In order to verify the accuracy of the ion current sensor for flame monitoring, an optical flame sensor with an ultraviolet phototube was used to synchronously monitor the flame with the ion current sensor. The signal intensity from the optical sensor could correctly reflect the flame state. The signal intensity of 0 V and 6 V referred to the flame extinction and combustion, respectively.

From Figure 17, the tendency of the ion current signal is consistent with that of the ultraviolet sensor signal. It confirms that the ion current sensor could correctly identify the flame ignition and extinction, and the sensor parameters determined in Section 4.1 are reasonable. Shapes of the ion current signals are similar and the typical shape can be divided into 3 sections: the section of the horizontal signal that corresponds to flame combustion, and the two sections of the step signals that refer to the flame ignition and extinction, respectively. For instance, at ${\dot{Q}}_{f}$ = 100 L/h, I stepped from 0 μA to 4.09 μA when the flame was ignited successfully; then I fluctuated around 4.09 μA along with flame combustion; finally, I dropped from 4.09 μA to 0 μA as the flame extinction.

**Figure 18:**
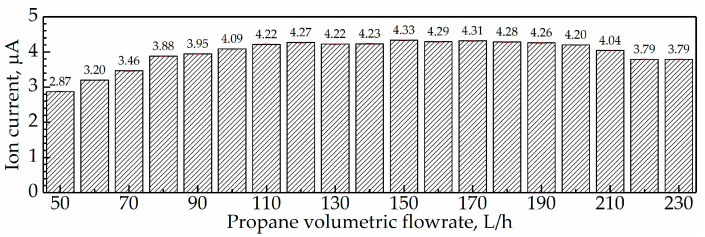
Average ion current during combustion at ${\dot{Q}}_{f}$ = 50–230 L/h.

In addition, the average ion current during flame combustion under ${\dot{Q}}_{f}$ = 50–230 L/h were acquired, and the results are displayed in Figure 18. In this Figure, the average I during combustion were all higher than 2 μA but lower than 5 μA.

## 5. Method of Ion Current Improvement and Verification for Flame Detection

### 5.1. Method of Ion Current Improvement

Provided that the electrodes have been adequately in contact with the flame, there are mainly two ways to greatly increase I, enhance $U_{e}$, and expand $S_{c}$.

The enhancement of $U_{e}$ requires a transformer, which brings about the problems of cost increase and weight increment that is especially harmful to aircrafts like fighters, and a high-voltage hazard that interferes with other electronic equipment. Thus, the method of improving the ionic current by greatly increasing $U_{e}$ is not feasible in industrial practice.

**Figure 19:**
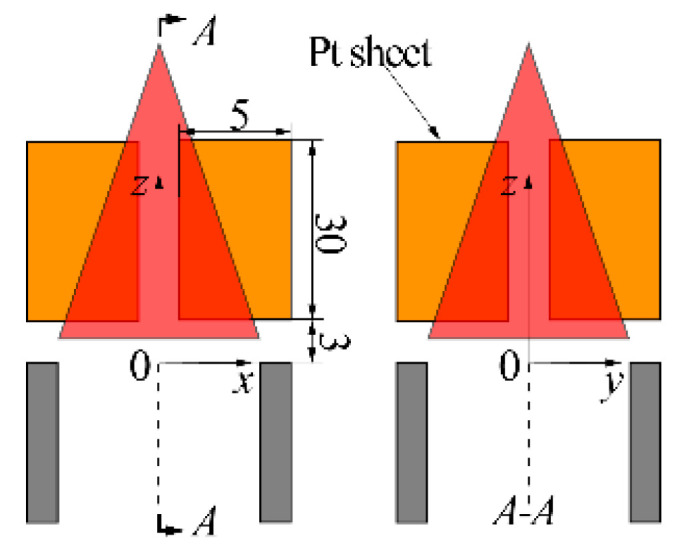
Schematic of the electrode structure with the added sheet cathode.

The expansion of $S_{c}$ that needs to add cathodes is a suitable measure to improve *I* in practical engineering. The specific structure proposed for expanding $S_{c}$ in this paper is shown in Figure 19. Four rectangular platinum sheets (5 mm × 30 mm × 0.1 mm) parallel to the radial direction of the burner exit were evenly arranged along the circumferential direction of the burner exit. The outer edges of the sheets and burner tube were aligned, which made the sheets contact adequately with the flame fronts under any circumstance. The lower edges of the sheets were 3 mm away from the tube exit, which ensures that the ignited flame could propagate continuously.

### 5.2. Verification of the Method for Ion Current Improvement

**Figure 20:**
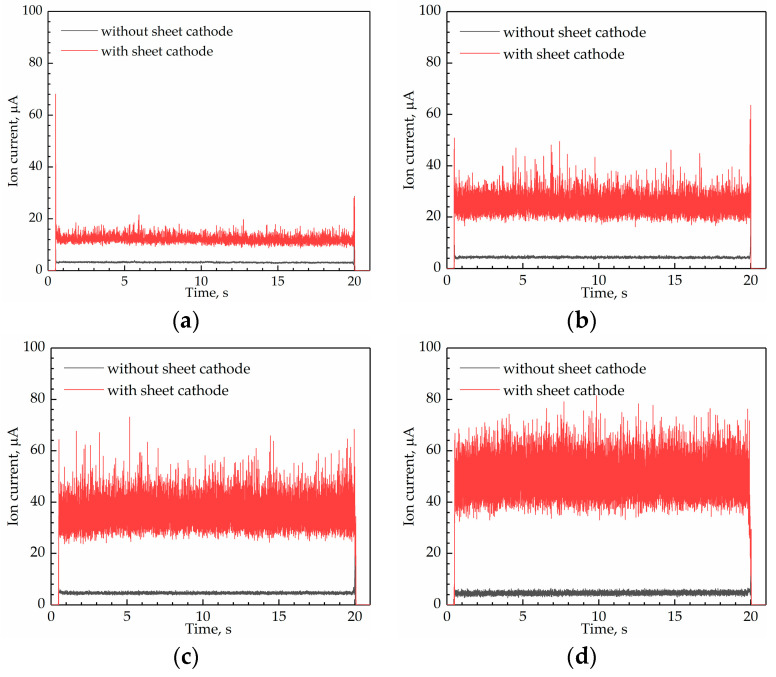
Comparison of the ion current signals from the original and improved ion current sensor: (**a**) ${\dot{Q}}_{f}$ = 50 L/h; (**b**) ${\dot{Q}}_{f}$ = 100 L/h; (**c**) ${\dot{Q}}_{f}$ = 150 L/h; (**d**) ${\dot{Q}}_{f}$ = 200 L/h.

The comparison of the I signals collected by the original (with the suggested sensor parameters) and improved (with the sheet cathode) sensors are displayed in Figure 20. The four subgraphs in the figure correspond to ${\dot{Q}}_{f}$ = 50 L/h, 100 L/h, 150 L/h, and 200 L/h. The black and red lines refer to the original and improved sensors, respectively.

From the figures, the signals from the improved sensor could correctly reflect the flame ignition, combustion, and extinction. Moreover, the great difference between the two signals was the amplitude of I. The amplitude of *I* collected by the improved sensor was higher and the detailed values of the average I during combustion are listed in Table 7.

**Figure 21:**
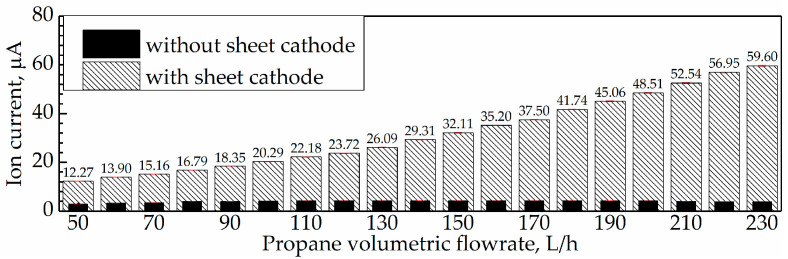
Schematic of the electrode structure for experiments of Factor 1.

In addition, the average *I* from the original and improved sensors during flame combustion of ${\dot{Q}}_{f}$ = 50–230 L/h were collected and the comparison between them is displayed in Figure 21. 

As for the original sensor, the I under ${\dot{Q}}_{f}$ = 50–230 L/h were all lower than 5 μA, and increased with ${\dot{Q}}_{f}$ ranging from 50 L/h to 150 L/h but decreased with ${\dot{Q}}_{f}$ ranging from 150 L/h to 230 L/h. As for the improved sensor, the I under ${\dot{Q}}_{f}$ = 50–230 L/h were all higher than 10 μA, and increased with ${\dot{Q}}_{f}$. Obviously, the improved sensor resulted in a higher ion current.

In summary, the method proposed by this paper to improve the ion current by expanding $S_{c}$ is effective.

## 6. Conclusions

In this work, a method for improving the ion current from the partially premixed flame on a Bunsen burner is proposed, to improve the reliability of the flame monitoring by the ion current sensor. The primary purposes of this work are (1) to investigate the effects of the ion current sensor parameters, including the excitation voltage, electrode area, and the electrode radial and vertical positions on the ion current; (2) to determine the reasonable sensor parameters for achieving a stronger and more stable ion current; and (3) to propose an effective measure to further strengthen the ion current.

The observed results are as follows. The ion current was linearly proportional to the excitation voltage. As for electrode area, if the electrode was inserted into the flame fronts, the ion current rose with the cathode area, whereas being constant with the anode area. As to the electrode radial position, the positions at which the electrode could be immersed into the flame fronts resulted in the higher ion current. As for the electrode vertical position, when the electrode was kept away from the flame base at a distance of at least 6 mm, the ion current was insensitive to the anode vertical position, yet very sensitive to the cathode vertical position. Correspondingly, to achieve a stronger ion current, the parameters of the ion current sensor used for flame monitoring on a Bunsen burner were suggested as follows. The excitation voltage should be set as 120 V, which was the maximum voltage applied in this work. The platinum wire should serve as the anode, while the burner should act as the cathode. The anode radial position should be 0 mm, at which the anode was entirely immersed into the flame fronts. The anode’s vertical position should be 6 mm away from the burner exit, which means that the anode was away from the flame base. A sensor with the recommended parameters was used in this experiment for flame monitoring on a Bunsen burner. The ion current signals from the sensor testified that the ion current could correctly signify the flame ignition, combustion, and extinction. Therefore, the suggested parameters are considered to be reasonable.

In addition, according to the regularities of the ion current varying with the sensor parameters, a method of adding a sheet cathode was proposed to further improve the ion current. Verification of the proposed method was performed during flame monitoring, and the results obtained shows that the proposed method of adding a sheet cathode had no effect on the responses of the ion current sensor to flame ignition and extinction, and significantly strengthened the ion current during combustion. Thus, adding a cathode area is an effective measure to improve the flame ion current.

## Figures and Tables

**Table 1:** Performance parameters of the instruments.

Parameter	Amplifier	Acquisitor
Manufacturer	Donghua, Jingjiang, China	
Model	DH3842	DH5939
Gain Accuracy	0.5%·FS	/
Measuring Accuracy	/	0.3%·FS

**Table 2:** Factors influencing the ion current.

Factor Number	Factor Name	Factor Notation
0	Electrode polarity	/
1	Excitation voltage	$U_{e}$
2	Electrode area	$S_{e}$
3	Electrode radial position	$\Delta_{2}$
4	Electrode vertical position	$\Delta_{1}$

**Table 3:** Experimental conditions for Factor 1.

Parameter	Range	Step
${\dot{Q}}_{f}$, L/h	100	/
$\Delta_{1}$, mm	5	/
$n_{e}$	1~5	2
$U_{e}$, V	10~120	10

**Table 4:** Experimental conditions for Factor 2.

Parameter	Range	Step
${\dot{Q}}_{f}$, L/h	100	/
$U_{e}$, V	120	/
$\Delta_{1}$, mm	5~53	12
$n_{e}$	1~5	1

**Table 5:** Experimental conditions for Factor 3.

Parameter	Range	Step
${\dot{Q}}_{f}$, L/h	100	/
$U_{e}$, V	120	/
$\Delta_{1}$, mm	5~65	15
$\Delta_{2}$, mm	0~20	1

**Table 6:** Experimental conditions for Factor 4.

Parameter	Range	Step
*U*_e_, V	120	/
$\Delta_{2}$, mm	0	/
${\dot{Q}}_{f}$, L/h	60~140	40
$\Delta_{1}$, mm	1~80	1

**Table 7:** Comparison of the average ion current from the original and improved sensor.

Sensor	50 L/h	100 L/h	150 L/h	200 L/h
Original	2.87 ± 0.015 μA	4.09 ± 0.015 μA	4.33 ± 0.015 μA	4.20 ± 0.015 μA
Improved	12.27 ± 0.06 μA	20.29 ± 0.15 μA	32.11 ± 0.15 μA	48.51 ± 0.15 μA
